# An Approach to an Anterior Shoulder Dislocation Using an Open Glenoid Stabilization With a Clavicle Autograft Compared to Other Interventions

**DOI:** 10.7759/cureus.78524

**Published:** 2025-02-04

**Authors:** Kevin T Dao, Youssef Messiha, Edvard Davtyan, Amritpal Dhillon, Mia Yasonova, Najib Ussef

**Affiliations:** 1 Internal Medicine, UCLA-Kern Medical, Bakersfield, USA; 2 Orthopedic Surgery, UCLA-Kern Medical, Bakersfield, USA

**Keywords:** anterior shoulder dislocations, bankart repair, clavicle autograft, latarjet, open glenoid stabilization, remplissage

## Abstract

The majority of joint dislocations tend to be shoulder dislocations, with the anterior shoulder dislocation being the most common. However, despite how common these types of injuries are, there have been frequent discussions regarding the type of treatment patients should receive and which surgeries are ideal to prevent recurrence. Some of the few surgeries include Bankart repair, remplissage, Latarjet procedures, and other glenoid stabilization surgeries. Here, we would like to present a patient who had an anterior shoulder dislocation, resulting in the patient having a remplissage with Bankart repair done at an outside institution, which failed. The patient then underwent an open glenoid augmentation procedure using the distal clavicle autograft rather than the traditional Latarjet procedure with a successful outcome. A discussion will also be held when comparing the majority of orthopedic procedures to prevent further anterior shoulder dislocations.

## Introduction

Shoulder dislocations are a common type of major joint dislocation, with an approximate incidence of 23.9 per 100,000 persons/year [1]. Anterior shoulder dislocations tend to be the most common, accounting for approximately 95% of cases [2]. These dislocations tend to occur due to some form of trauma resulting in an externally rotated and abducted arm. In many instances, physicians should always assess for any nerve damage, particularly the axillary nerve, although in some situations, a simple reduction can resolve abnormal motor functions [2]. Other complications can occur, but supportive measures are initially ideal, which involve reduction and immobilization along with physical therapy. In fact, many studies have discussed various types of reduction techniques as well as their success rate [3-4]. Despite this, recurrent dislocations can still arise, and in many situations, patients with a prior history of shoulder dislocations can relapse. Therefore, many surgical options are available for patients to prevent recurrent shoulder dislocations. The more common surgical interventions include, but are not limited to, arthroscopic versus open Bankart Repair, which can be combined with remplissage, as well as open glenoid augmentation such as a Latarjet, or augmentation using the distal tibial allograft or iliac crest autograft [5-6]. In fact, many studies have portrayed high levels of success with patients who have done a Bankart with remplissage when compared to the Latarjet procedure or the Bankart procedure separately [6]. In some instances, there have been previous procedures of using the distal clavicle in order to augment the glenoid [7]. In those cases, arthroscopic measures have been employed; however, this in and of itself can be very technically challenging when compared to the open approach. As such, we present a patient who had an anterior shoulder dislocation requiring an open glenoid augmentation procedure and who had failed Bankart with remplissage. What is unique about this case is that the clavicle was used as an autograft rather than the coracoid given the patient had a preexisting coracoid fracture, and unfortunately, the distal tibial allograft is not readily available at our institution. A discussion and review will be held regarding these various types of surgical interventions as well.

## Case presentation

The patient is a 27-year-old man with a past medical history of a motor vehicle accident in 2014 that resulted in multiple shoulder dislocations. He notes that these dislocations required frequent emergency department reduction, prompting the patient to establish care with an orthopedic physician for a longer-lasting solution. The patient underwent left arthroscopic Bankart repair with remplissage at an outside institution. Unfortunately, the patient sustained five subsequent dislocations after his stabilization procedure. All the other medical history was unremarkable.

**Figure 1:**
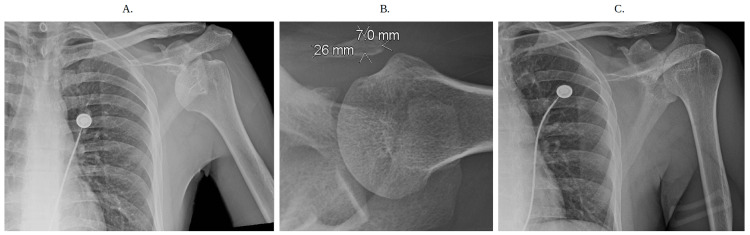
Initial X-rays of the left shoulder at the emergency department on presentation (a) There is a partial anterior and inferior dislocation of the humeral head without fracture with the acromioclavicular joint which is maintained. (b) There is a 2.6 × 0.7 cm corticated osseous density in the anterior region of the shoulder which may be due to old trauma. (c) Figure shown displays proper reduction of the dislocated left humeral head

**Figure 2:**
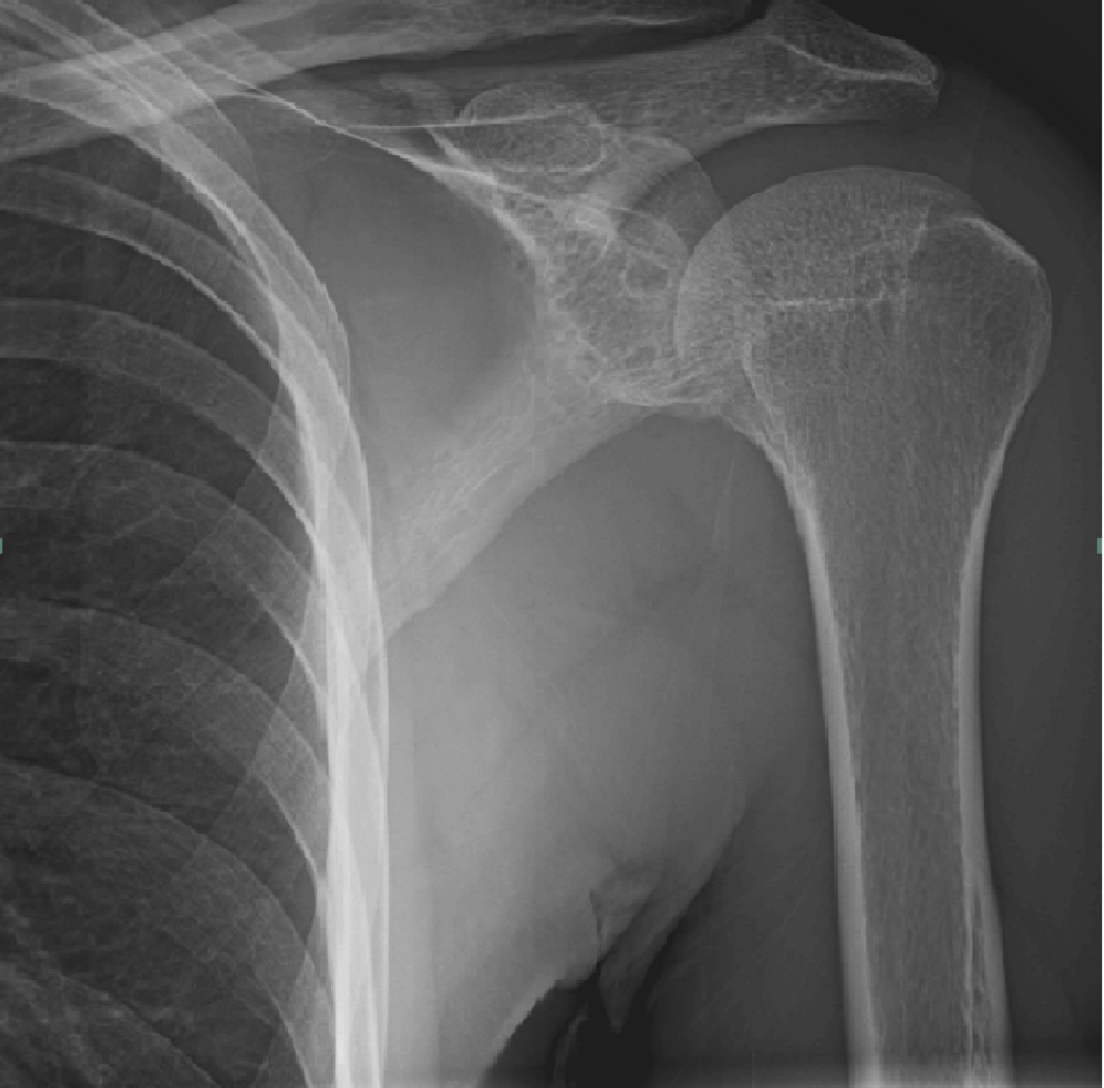
Repeat left shoulder X-ray The acromioclavicular and glenohumeral joints remain in proper anatomic alignment

He presented to the orthopedic clinic a few weeks after being referred from the emergency department for postreduction due to another left shoulder dislocation while doing activities of daily living (Figures 1a-1c). On the physical exam, the patient was noted to have a full shoulder range of motion with anterior apprehension and relocation. In addition, the patient demonstrated mid-range apprehension at 80-90 degrees of forward flexion. His Beighton score was 0. Shoulder radiographs at initial evaluation demonstrated a large Hill-Sachs lesion with a well-reduced glenohumeral joint (Figure 2).

**Figure 3:**
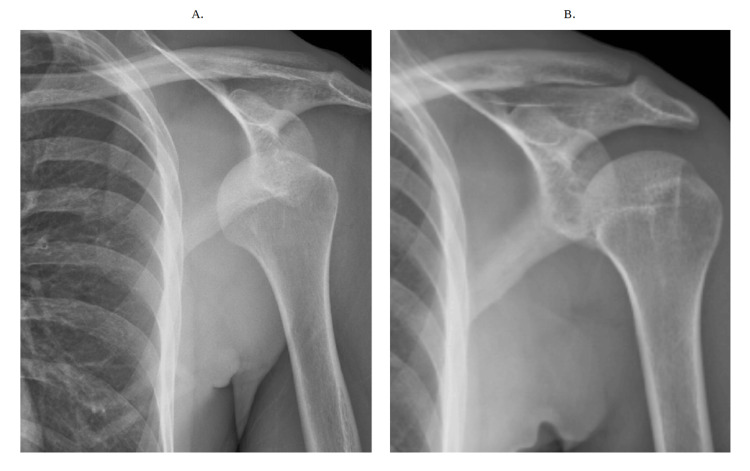
Repeat X-ray of the shoulder shows a dislocation of the left shoulder (a) Anterior shoulder dislocation is present with some re-demonstration of exostosis arising from the coracoid process and well-corticated ossification anterior to the shoulder. (b) Proper interval reduction of anterior shoulder dislocation

**Figure 4:**
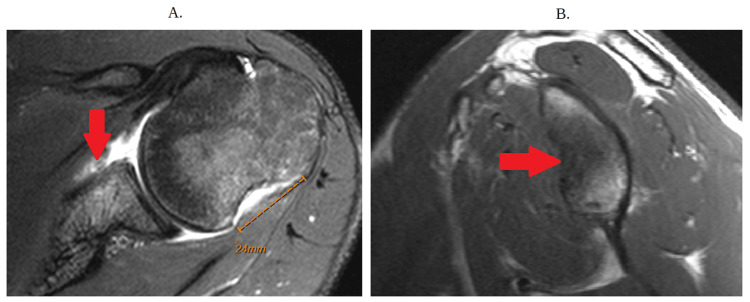
Magnetic resonance imaging without contrast (a) The arrow depicts the labral tear. A large Hill-Sachs lesion of approximately 24 mm indicating evidence of failed remplissage is also noted. (b) The red arrow depicts bone loss

**Figure 5:**
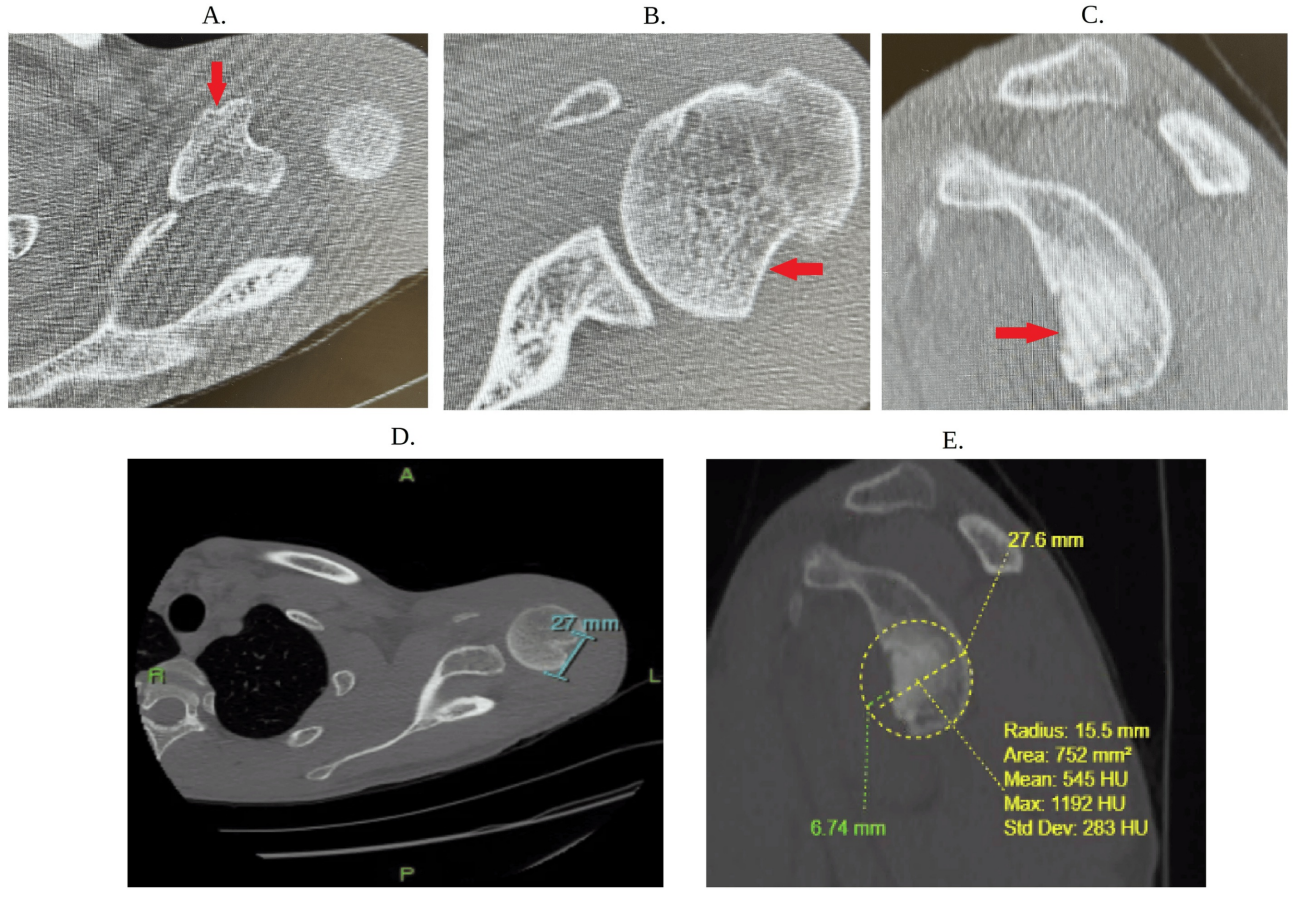
CT scan (a) The red arrow indicates the fractured coracoid. (b) The red arrow indicates the Hill-Sachs lesion. (c) This view is a sagittal CT view with the arrow showing bone loss. (d) Glenoid track measurement was based on the horizontal diameter of the glenoid, which is approximately 27 mm. The glenoid track calculation (0.83 × 27 mm)-6 mm shows a glenoid bone loss of approximately 16 mm. (e) Based on the measurement of the Hill-Sachs interval and glenoid track provided using the best-fit circle method in yellow, the Hill-Sachs interval is approximately 33 mm with magnetic resonance imaging without contrast in Figure 4 of approximately 24 mm, which is still greater than the glenoid track bone loss of approximately 16 mm compatible with an engaging off-track Hill-Sachs defect

An appointment was made for further MRI without contrast and CT scan without contrast with 3D reconstruction to further evaluate the intraarticular structures for further management; however, the patient unfortunately had another dislocation shortly after, and another reduction was done (Figures 3a-3b). An MRI without contrast was done, which showed a large impacted fracture of the anterior glenoid associated with a torn anterior labrum, and an impacted fracture (Hill-Sachs lesion) was noted at the humeral head (Figures 4a-4b). A CT scan was also done (Figures 5a-5c), and at the follow-up appointment, the patient expressed interest in another shoulder stabilization surgery.

A discussion was held with the patient, and it was decided to do left shoulder glenoid reconstruction with a left distal clavicle autograft rather than a traditional Latarjet procedure, given the patient had a chronic appearing coracoid fracture on CT scan (Figures 5a-5c). A distal tibial allograft was also considered; however, we do not have timely access to these grafts at our institution.

Procedure details

**Figure 6:**
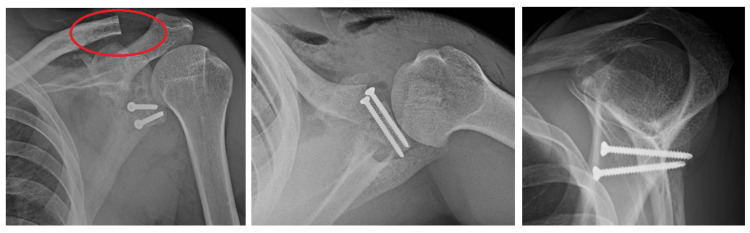
Repeat X-ray after glenoid reconstruction The red circle noted is where there is a distal clavicular resection. The rest of the figures show the internal fixation of the anterior glenoid defect with two threaded screws

A standard deltopectoral approach was utilized, except the incision was extended proximally by 3 to 4 cm toward the acromioclavicular (AC) joint. The coracoid fracture was first identified and was chronic in appearance with degeneration of the remaining fragment. Of note, a subscapularis sparing approach was used, and the subscapularis tendon was split longitudinally and the lower two-thirds in line with its fibers, and a T capsulotomy was performed. The fragment was excised, and the conjoined tendon was tagged with #2 FiberWire, and plans for later conjoined tendon transfer. Attention was then turned to the distal clavicle autograft harvest. Dissection was performed down to the deltotrapezial fascia and AC joint capsule, which was incised in line with the clavicle. Care was taken to elevate full-thickness flaps anteriorly and posteriorly to allow for meticulous repair of the AC joint capsule and deltotrapezial fascia at the end of the case. Once the distal clavicle was identified, small Hohmann retractors were placed anteriorly and posteriorly, and an Army-Navy retractor was placed laterally. A point 11 mm from the distal aspect of the clavicle was then measured and marked using electrocautery in line with the planned resection. An 11 mm rather than 10 mm was marked to account for the kerf of the saw. We used a small 10 mm oscillating saw to perform the resection. The resected distal clavicle autograft was then carefully removed in its entirety using electrocautery to dissect away any remaining soft tissue attachments. Great care was taken not to cause a fracture or fragmentation of the autograft during its removal. The graft was then placed into 1 g of vancomycin powder mixed with 200 cc of normal saline. In terms of the orientation of the graft onto the anterior glenoid, multiple options were considered, including using the cancellous aspect of the graft to place onto the anterior glenoid surface versus using the inferior or superior surface of the clavicle so that the cartilaginous aspect of the distal clavicle would articulate with the humeral head. For this particular case, a decision was made to use the cancellous side of the graft in order to increase bony healing and to create the largest height of the graft based on measured dimensions. The anterior glenoid was then prepped as usual using a curette and osteotome. There was about 30% measured glenoid bone loss. A decision was made to use 3.5 mm fully threaded solid cortex screws for fixation of the graft. The graft was predrilled with a 3.5 mm drill in preparation for the placement of the lag screws. The anterior glenoid was then predrilled with a 2.5 mm drill in the region of the planned graft placement. Only the superior drill hole was drilled. The first 3.5 mm screw was then preliminarily placed into the graft and then advanced into the drill hole that was drilled onto the anterior glenoid to secure the graft in place. The offset of the graft was checked in relation to the anterior glenoid to ensure there was no overhang. The second, more inferior glenoid drill hole was then made, and the second screw was placed, and excellent fixation was obtained. Although there are some risks of graft breakage with screw placements, it is fairly uncommon, and usually, bigger screws, such as 4.5 mm, are more common since larger holes are required for bigger screws. An all-suture double-loaded anchor was then placed onto the anterior glenoid, and the suture strands were passed through the conjoined tendon and tied to secure transfer of the conjoined tendon onto the anterior glenoid in order to provide a further sling effect and further stabilization. Standard closure of the capsule and deltopectoral interval was performed with 0 vicryl. The patient was placed in a shoulder abduction sling for four weeks. On the follow-up appointment, the patient was doing well, and an X-ray demonstrated a stable glenoid reconstruction with a distal clavicle autograft (Figure 6).

## Discussion

Bankart repair procedures are common surgeries geared toward repairing the labrum in dislocated shoulders. Remplissage procedures are techniques that attempt to fill in Hill-Sach defects by suturing the infraspinatus tendon and posterior capsule into the Hill-Sach defect. A Hill-Sachs lesion is a bony impaction/indentation and/or fracture at the superior portions of the humerus that occurs when the shoulder dislocates anteriorly with a deeper and larger lesion, resulting in more shoulder instability. In some instances, orthopedic surgeons have combined both of these techniques into a Bankart with remplissage, and such surgeries have shown quite a bit of success [8]. Latarjet procedures attempt to restabilize the damaged shoulder socket by autografting the coracoid bone to the area to prevent reoccurring dislocations.

Glenoid augmentation refers to procedures that attempt to stabilize the shoulders in patients with recurrent shoulder instability due to bone loss via an autograft or allograft and can be done with anterior or posterior bone loss, with the former being more common. Glenoid augmentation autograft options have been utilized in recent years, including the iliac crest autograft and the distal clavicle autograft. In addition, multiple allograft options have also been used, most commonly the distal tibial allograft. To our knowledge, no previous reports or techniques have been done on an open distal clavicle autograft glenoid augmentation procedure. There have been multiple technique articles and studies performed on using the distal clavicle autograft through an arthroscopic technique, which have provided excellent results [7]. This technique can be technically challenging for some surgeons who more commonly perform open glenoid augmentation procedures. We hope that this case may provide an additional open glenoid augmentation option that can be done through the same incision. The iliac crest autograft option is also a good option but may result in graft harvest site complications such as chronic pain or hematoma. We do believe that the Latarjet or coracoid transfer is the gold standard for open stabilization procedures and is the most cost-effective. However, there may be certain cases or situations where a coracoid transfer cannot be performed, such as this case where the patient had a preexisting coracoid fracture since it would be unwise to use a graft of a bone that has already been fractured. Other cases may include a patient with a failed Latarjet procedure. We also believe that there are certain situations where preserving the coracoid may be advantageous. The situations include slightly older patients with recurrent shoulder instability and significant glenoid bone loss, where the surgeon may be concerned about future glenohumeral arthritis formation. We do believe that converting a Latarjet to a total shoulder is technically more challenging than converting other glenoid augmentation procedures to a total shoulder due to the alteration in anatomy and the higher risk for neurovascular injury. In regard to neurovascular injury, we do believe that preserving the coracoid and using other bone augmentation procedures may potentially have a lower risk of nerve injury.

Multiple studies have shown various comparisons between these surgeries in order to establish which surgical intervention is ideal for the patient. When comparing isolated arthroscopic Bankart repair with Latarjet, Latarjet procedures show lower dislocation rates but higher complication rates. In fact, one study noted that patients who underwent an open Latarjet procedure had fewer recurrent dislocations when compared to the open or arthroscopic Bankart group [9-10]. Another study also compared open Latarjet procedures to open Bankart procedures, and the results show that the radiological outcomes and stability of the shoulders of the athletes examined depicted the superiority of open Latarjet procedures [11]. One study noted that for off-track Hill-Sachs lesions, there were greater complications that arose in the Latarjet group. Yet, the same study also noted that Latarjet procedures are a superior choice for patients with greater than 10% glenoid bone loss in patients with frequent shoulder instability [12]. When it comes to Bankart repair with remplissage versus Latarjet, there is an ongoing debate on which procedure is most appropriate for different degrees of bone loss. One study concluded that in patients who had a 10 mm or more Hill-Sachs depth lesion, the degree of recurrent instability was approximately equal when comparing Bankart repair with remplissage versus Latarjet. In fact, the study concluded that patients who underwent a Latarjet had a superior range of motion in their repaired shoulder [13]. Overall, Latarjet procedures tend to be quite reliable surgeries in patients with recurrent shoulder instability, but the question remains whether an arthroscopic Latarjet is as good as an open Latarjet. A systemic review asked the same question and compared open Latarjet to arthroscopic Latarjet. The study showed that there is a much higher cost associated with arthroscopic procedures, with few overall benefits [14-15]. In terms of cost-effectiveness, another retrospective review determined that after 90 days there was no significant difference in the complication rates when comparing open to arthroscopic Latarjet procedures and highlighted some of the downsides of an arthroscopic Latarjet, such as the technicality of mastering the procedure [16].

This goes to show that when it comes to anterior shoulder dislocations with significant bone loss, open procedures are not only possible but maybe even preferable. However, this doesn’t mean that other orthopedic procedures should be neglected. It should be noted that orthopedic physicians should keep in mind the potential that open Latarjet procedures can have when compared to other surgical shoulder stabilizing procedures. When it comes to open glenoid stabilization, this case shows that the distal clavicle can be successfully used during a glenoid augmentation procedure. However, this should be done on a case-by-case basis. In patients with higher glenoid bone loss, it is better to perform glenoid augmentation where subcritical bone loss is around 10%; however, this would be considered debatable if patients had increased risk factors such as contact sports. With regard to 15%-20% glenoid bone loss, glenoid augmentation generally has better long-term outcomes, especially with regard to dislocation rates. In regard to patients that have failed prior procedures of having 25%-30%, distal tibial allograft and iliac crest autograft should be considered. In addition, we do believe that distal clavicle autograft can also be considered in these cases of higher glenoid bone loss.

This raises an important question regarding how the glenoid bone loss helped determine the severity of the patient’s shoulder instability. In short, the glenoid track is an area of contact between both the glenoid and the humeral head. In normal patients, the contact is approximately 83% of the width of the glenoid. Thus, a patient with a Hill-Sachs defect that is smaller than the track will maintain proper contact and will be “on track,” resulting in less instability and vice versa. Our patient was noted to be both “off track,” with the glenoid track calculation of approximately 16 mm based on the calculation (0.83 × 27 mm)-6 mm (Figure 5d). In both Figure 4a and Figures 5a-5e, the patient was noted to have a Hill-Sachs of 24 mm on MRI and 33 mm on CT scan, which are both greater than the glenoid, meaning that regardless of MRI or CT scan, the patient had shoulder instability.

Overall, this should be based on surgeon preference and individualized to each patient’s situation. Another unique point to make is that, in some instances, patients can also have fractures of the coracoid, and prior techniques can use the distal iliac crest; however, this case shows that the clavicle is another area where an autograft can be used, and an open approach can be done rather than an arthroscopic one. This case truly shows that when compared to the traditional arthroscopic procedures, there is flexibility to be had, and in instances where surgeons who don’t have experience with arthroscopic augmentation procedures should consider an open approach can be done using the clavicle.

## Conclusions

Overall, anterior shoulder dislocations are a very common occurrence, especially after physically traumatic events. While there are dozens of different reduction techniques, complications such as recurrent dislocations can still occur, and surgical intervention might be warranted. As such, many options are made available to patients with anterior shoulder dislocations. Procedures can be open to arthroscopic and can involve Bankart repair, remplissage, Bankart with remplissage, Latarjet, or other glenoid stabilization procedures. Although this is not an extensive list, this case report does show that even if one surgery fails, another type of repair can be warranted. The truly interesting detail regarding this case, however, is that the patient was able to tolerate an open glenoid procedure using the clavicle autograft. Although this is just one instance, it should be made clear that further, larger studies should be done to validate this approach. Regardless, hopefully, this case can help provide flexibility for orthopedic physicians who are unable to use the coracoid for different reasons and would want to go with an open approach with the option of using the clavicle.
